# A new species of *Chalastonepsia* Søli, 1996 (Diptera, Mycetophilidae) from Vietnam

**DOI:** 10.3897/BDJ.13.e153645

**Published:** 2025-08-07

**Authors:** Svetlozara Kazandzhieva, Mario Langourov, Dimitar Bechev, Hieu Van Nguyen

**Affiliations:** 1 National Museum of Natural History, Bulgarian Academy of Sciences, Sofia, Bulgaria National Museum of Natural History, Bulgarian Academy of Sciences Sofia Bulgaria; 2 Department of Zoology, University of Plovdiv, Plovdiv, Bulgaria Department of Zoology, University of Plovdiv Plovdiv Bulgaria; 3 Hanoi Pedagogical University 2, Phu Tho, Vietnam Hanoi Pedagogical University 2 Phu Tho Vietnam

**Keywords:** Diptera, Sciaroidea, Gnoristinae, Metanepsiini, Oriental, fungus gnats, taxonomy, new taxon

## Abstract

**Background:**

The genus *Chalastonepsia* Søli, 1996 was erected for the species *Chalastonepsiaorientalis* Søli, 1996 from Pahang, Peninsular Malaysia. The species *C.hokkaidensis* Kallweit, 1998 was described from Hokkaido, Japan. Ševčík and Hippa (2010) described two additional species of *Chalastonepsia*: *C.nigricoxa* Ševčík & Hippa, 2010 from Sumatra and Peninsular Malaysia and *C.montana* Ševčík & Hippa, 2010 from N.W. Thailand.

**New information:**

A new Oriental species is described in the genus *Chalastonepsia* of the subfamily Gnoristinae – *Chalastonepsiavumanhi* Kazandzhieva sp. nov. from a single locality in Tam Dao National Park in northern Vietnam. The new species is closely related to *C.hokkaidensis* Kallweit, 1998, but differs from it by the number of long anterior projections on flagellomeres (1–9), the shape and size of T9, details on the gonostyli and their appendages. An identification key to the extant species of *Chalastonepsia* is provided. The taxonomy of the genus *Chalastonepsia* is shortly discussed in relation to the distinct morphology of the type species *C.orientalis* Søli, 1996.

## Introduction

The tribe Metanepsiini (treated as subfamily by some authors, for example, Väisänen (1984), Väisänen (1986) and Ševčík and Hippa (2010) was established by Matile (1971) for three species of *Metanepsia* Edwards, 1927 (Edwards 1927). Kallweit (1998) noted that the delimitation of the Metanepsiini against Gnoristinae (sensu Väisänen (1986)) is obscure and a clarification of its position within Mycetophilidae is awaiting a revision of the Gnoristinae. As a result of recent molecular studies, Metanepsiinae was shown to belong to Gnoristinae and not to form a monophyletic clade (Ševčík et al. 2013, Kaspřák et al. 2019). Oliveira and Amorim (2012) question the subfamilial rank of Metanepsiini and argue that it consists a smaller clade than Gnoristinae, thus, without a phylogeny of the subfamily, it would render the Gnoristinae paraphyletic. Currently, the systematic position of the Metanepsiini is uncertain; hence, in this study, we use the term Metanepsiini tentatively for the three genera *Metanepsia* Edwards, 1927, *Chalastonepsia* Søli, 1996 and *Pectinepsia* Ševčík & Hippa, 2010.

*Metanepsia* comprises nine described species, two of which are Oriental: *M.javana* Edwards, 1927 described from Indonesia and *M.malaysiana* Kallweit, 1998 described from Peninsular Malaysia and seven Afrotropical species described by Matile (Matile (1971), Matile (1972), Matile (1975) and Matile (1980). The genus is also known from Thailand (Ševčík et al. 2013, Kaspřák et al. 2019), Brunei and Vietnam (Kaspřák et al. 2019). The genus *Chalastonepsia* was erected for the species *C.orientalis* Søli, 1996 from Pahang, Peninsular Malaysia (Søli 1996). The species *C.hokkaidensis* was described by Kallweit (1998) from Hokkaido, Japan. Ševčík and Hippa (2010) described two additional species of *Chalastonepsia*: *C.nigricoxa* from Sumatra and Peninsular Malaysia and *C.montana* from N.W. Thailand, as well as a new genus in the tribe – *Pectinepsia* Ševčík & Hippa, 2010 for the species *P.pulcherrima* from Sarawak, Borneo and *P.sumatrensis* from Sumatra. The genus *Chalastonepsia* was reported also from Brunei (Ševčík and Hippa 2010, Kaspřák et al. 2019), Taiwan, Indonesia and Papua New Guinea (Ševčík and Hippa 2010), suggesting a wide distribution in the Oriental Region and beyond.

In the present article, a fifth species of *Chalastonepsia* is described and illustrated from a single locality in Tam Dao National Park in Vietnam. The specimen described below is in the collection of the National Museum of Natural History – Sofia.

## Materials and methods

**Figure 1.**
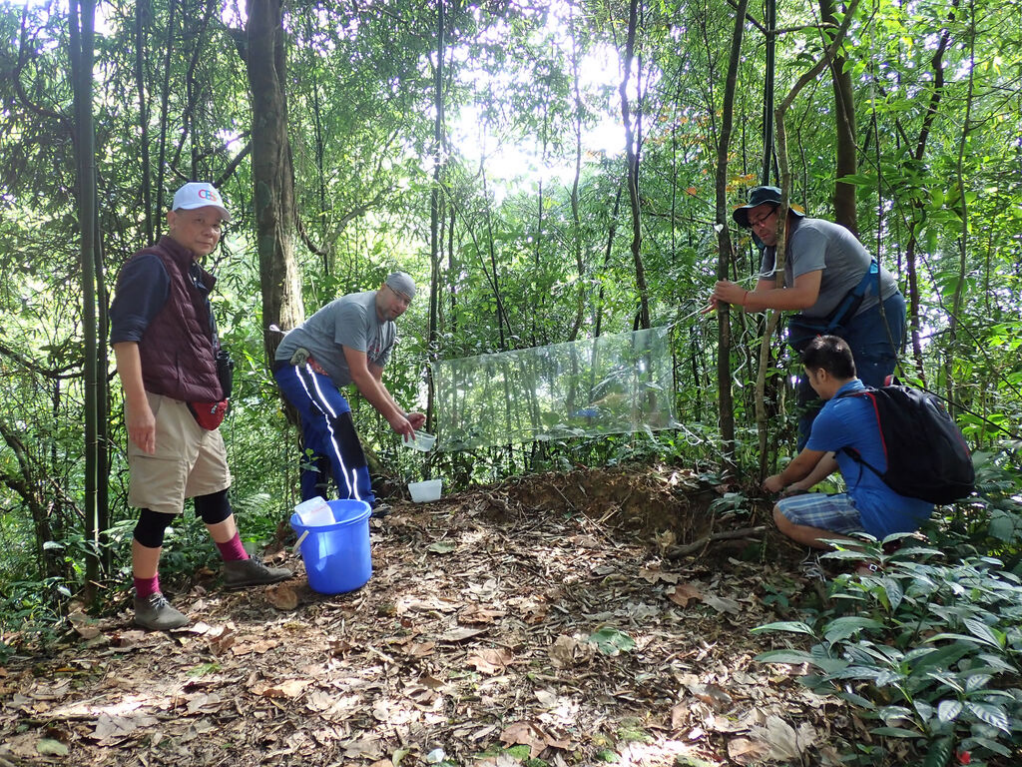
Habitat and collection method of *Chalastonepsiavumanhi* sp. nov. From left to right Manh Quang Vu, Rostislav Bekchiev, Mario Langourov and Hieu Van Nguyen.

The studied material was collected by Rostislav Bekchiev, Mario Langourov and Nikolay Simov by flight interception traps in a lower mountain dipterocarp forest habitat near Chùa Vàng around the village of Tam Đảo (Fig. 1) and was preserved in ethanol. The habitus photos (specimen in alcohol) were taken by digital camera Canon EOS 2000D fitted to a Carl Zeiss Stemi 2000 stereomicroscope. The terminalia of the specimen were removed and subsequently macerated in 10% warm potassium hydroxide (KOH). Dissections and temporary slides were made in glycerol. The slides were photographed with a Canon EOS 2000D fitted to a Carl Zeiss Jena Amplival microscope. Terminalia were afterwards transferred to microvials with glycerine and stored together with the specimen. The habitus and male terminalia photos were combined using Helicon Focus 7 software from multiple gradually focused images. The morphological terminology follows Søli (1997) and Søli (2017). The studied material is deposited in the collection of the National Museum of Natural History, Sofia (NMNHS).

## Taxon treatments

### Chalastonepsia vumanhi

Kazandzhieva sp. nov.

437EEE17-30DC-5621-8A87-4D2B3352084B

D3116317-ACA4-4F52-9CD8-D98EE65FC0DB

#### Materials

- **Type status:** Holotype. **Occurrence:** catalogNumber: BG-NMNHS-ENT-000000019865; recordedBy: Svetlozara Kazandzhieva; individualCount: 1; sex: male; lifeStage: adult; preparations: In ethanol; occurrenceID: 23E47D56-7971-56E6-ACE0-48E29A57875C; **Taxon:** scientificName: Chalastonepsiavumanhi; order: Diptera; family: Mycetophilidae; genus: Chalastonepsia; specificEpithet: vumanhi; scientificNameAuthorship: Kazandzhieva; **Location:** country: Vietnam; stateProvince: Phu Tho; verbatimLocality: Tam Dao NP, Tam Đảo, ab Chùa Vàng Tam Đảo; verbatimElevation: 1067 m; verbatimLatitude: 21° 27.6348' N; verbatimLongitude: 105° 38.9184' E; verbatimSRS: WGS84; decimalLatitude: 21.46058; decimalLongitude: 105.64864; **Identification:** identifiedBy: Svetlozara Kazandzhieva; dateIdentified: 2024; **Event:** samplingProtocol: flight interception trap; eventDate: 16-10-2023; habitat: lower mountain dipterocarp forest; **Record Level:** collectionID: BG-NMNHS-ENT-000000019865; institutionCode: NMNHS; collectionCode: Insects; basisOfRecord: PreservedSpecimen

#### Description

**Male** Body length 2.4 mm

**Figure 2.**
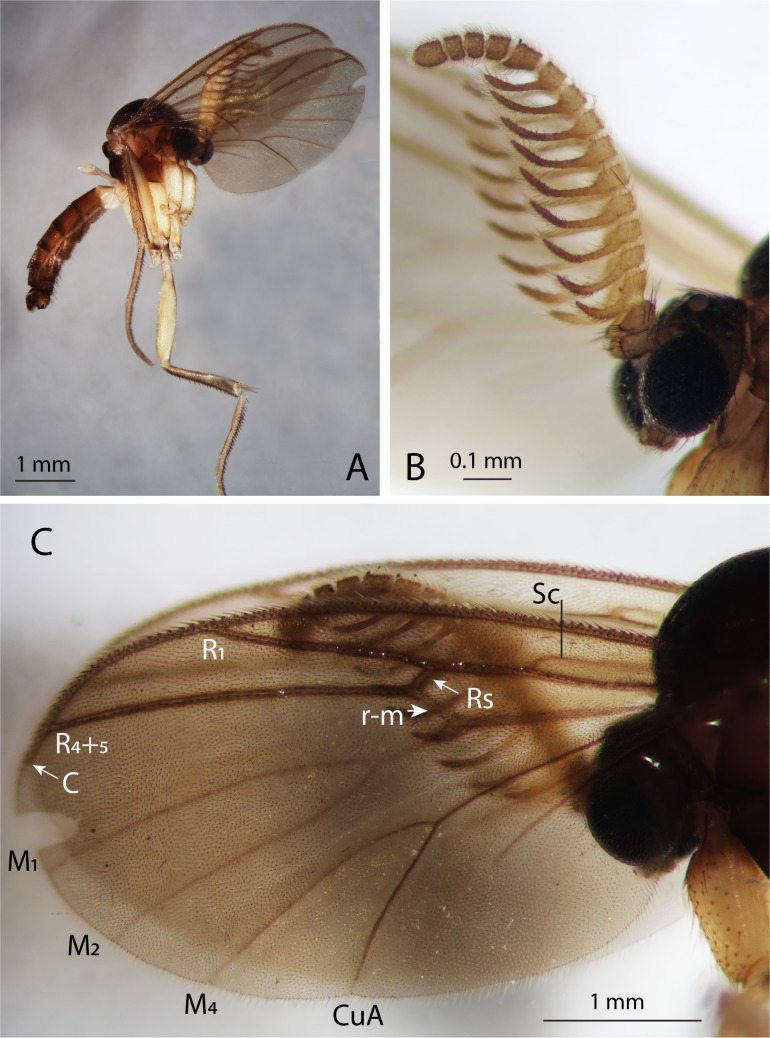
*Chalastonepsiavumanhi* sp. nov. holotype, male (BG-NMNHS-ENT-000000019865). **A** habitus; **B** antennae; **C** wing. Scale bars: 1 mm (A, C); 0.1 mm (B).

**Head** (Fig. 2B): Head mostly dark brown, palpi, labella and occiput paler. Antennae yellow; scape, pedicel and the apical five flagellomeres slightly darkened. Ocelli three, virtually in one line, the median one about half the diameter of each lateral one, separated from eye margin and the median ocellus for a distance of about 1.5 times their own diameter. Eyes emarginate above base of antennae, covered with fine hairs. Frons bare. Face densely bristled, shield-like. Clypeus bare, rectangular, longer than broad, not longer than face. Mouthparts reduced, setose, with one visible palpomere. Antennae strongly pectinate, with 14 flagellomeres. Flagellomeres 1–9 each with long anterior projections (Fig. 2B). Flagellomere 10 only slightly extended, projection about half its diameter. Flagellum mostly light yellow, last 5 flagellomeres darker, flagellar projections apically darkened. Flagellomeres 11–14 not modified. All flagellar segments covered in fine setae about as long as the diameter of the projections apically. Scape and pedicel with some stronger short anterior bristles and less, but longer posterior bristles. Antenna about 1.2 times as long as thorax. Postcranium and genae bristled, with a distinct furrow between median ocellus and tip of the frontal tubercle. No suture present between eye margin and lateral ocellus.

**Figure 3.**
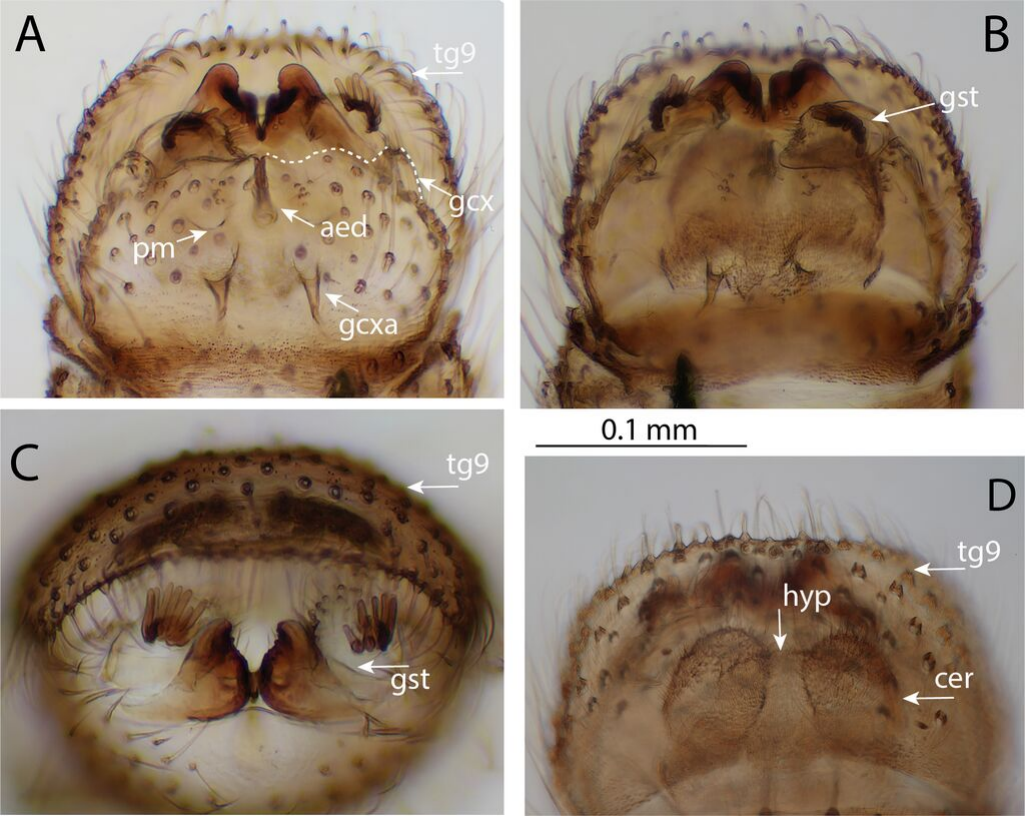
Male terminalia of *Chalastonepsiavumanhi* sp. nov. **A** ventral view of gonocoxal outline; **B** ventral view of gonostylus; **C** dorso-posterior view; **D** tergite 9 with hypoproct and cerci. Scale bars: 0.1 mm (A–C). Abbreviations: gcx = gonocoxite, aed = aedeagus, pm = paramere, gcxa = gonocoxal apodeme, gst = gonostylus, hyp = hypoproct, tg 9 = tergite 9, cer = cercus.

**Thorax**: Mostly dark brown; upper half of anepisternum paler, upper end of anepimeron light yellow, edges of the mediotergite paler. Anepimeral cleft distinct. Scutum evenly covered with relatively short setae (only three of them left, all the rest – lost), with two paler acrostichal stripes. No prescutal suture. Scutellum with a transverse row of 12–15 stronger setae (most of them lost) and with numerous weaker setae scattered over upper half. Mediotergite bare. Laterotergite with 12 setae medially, most of them lost. Anepisternum bare. Nine pro-episternal bristles present, 2–3 setae on anteprononutum. Pro-epimeron brown, bare, basisternum 1 light yellow, katepisternum brown, bare. Metanotum, metepimeron and metepisternum light yellow with no visible setae, except for one near base of halters. **Wings** (Fig. 2C): Length 2.3 mm, width 1.1 mm. Wing unmarked, hyaline, with dense irregularly arranged microtrichia, without macrotrichia, R_1_ and R_4+5_ with a line of setae above. Other postcostal veins without setae. C produced for about one third of the distance between R_4+5_ and M_1_, hardly reaching wing tip. Sc long, ending in R_1_ well before base of Rs. Rs distinct, about ¾ as long as crossvein r-m. M-branches reaching wing margin; M_1_ about five times (5.5 times) as long as M-petiole, M_2_ a little shorter. Point of furcation of CuA beyond base of crossvein r-m. M-petiole, M_1_, M_2_ and M_4_ weak; CuA more distinct. CuP faint, ending at about level of CuA-furcation, A_1_ stronger ending before CuA-furcation. Halteres pale yellow, whitish, covered with short hairs, very fine at apical part. **Legs**: Entirely yellow, except for a dark apical patch on inner side of fore- and mid-coxae and predominantly on inner side of hind coxae. Hind coxae with a complete vertical row of setae (8 or 9) along outer hind margin. Fore tibia as long as fore femur. Spurs yellow, covered in short fine hairs. Spur formula after Vockeroth (1980): 1.3; 1.25, 2.25; 1.2, 2. Antero-apical tibial organ of fore- and mid-tibia missing. Fine tibial and tarsal setae irregularly arranged, dark. Fore leg without strong bristles, except for 5–6 apically on tibia. Tibiae and tarsomeres 1–4 of mid- and hind leg with rather short, but distinct bristles, most of which arranged in lines, the longest about half as long as tibial diameter at apex. These bristles strongly modified, bearing 4–5 narrow leaf-like lateral processes (see fig. 8, Kallweit (1998)). Tarsal claws each with two larger and one smallеr ventral tooth. Empodium small. **Abdomen**: First segment pale yellow, other segments brown, subapically darker. Sternites 1 and 8 bare. Tergite 1, segments 2–7 and tergite 8 covered with dark setae, most of them lost. Sternites 2 to 6 each with a pair of pale yellow, well-defined, submedian lines. Sternite 6 with fold-lines on basal quarter only. Segments 6–8 reduced. **Terminalia** (Fig. 3A–C): Terminalia brown. Tergite 9 large, not fused with gonocoxites, about 1.8 times as wide as long; not rounded apically, straight, slightly concave; dorsally and apical quarter ventrally with setae. Cerci rounded, apically with few setae. Hypoproct rounded, not subtriangular, with 2–3 setae on either side of apex. Parameral apodemes long, curved outwards. Parameres with seven short setae near base. Parameres and aedeagus not clearly separable. Aedeagus stalk-like, elongate, narrow. Gonocoxites fused ventrally, with dark dense bristling, except for a bare median stripe. Posteromedially to gonocoxites, a pair of heavily sclerotised oval appendages, rounded apically with less distinct dent than in *C.hokkaidensis* (see fig. 11, Kallweit (1998)), each with three short median bristles. Gonostylus small, 1.7 times longer than wide, apically convex, setose basally, with eight distinct setae subapically before the protruding apical part; bearing a brush of around 9–10 finger-like thick setae.

**Female**. Unknown.

#### Diagnosis

*Chalastonepsiavumanhi* sp. nov. differs most conspicuously from the other representatives of the genus, by flagellomeres 1–9 with long anterior projection and details on the genitalia. The new species *C.vumanhi* sp. nov. is closely related to *C.hokkaidensis*, sharing the complex structure of the genitalia, but differing in the number of long anterior projections on flagellomeres (*C.hokkaidensis* 1–10, *C.vumanhi* sp. nov. 1–9), in the shape of T9 (in *C.hokkaidensis* apically rounded and slightly tapering, in *C.vumanhi* sp. nov. straight and slightly concave – Fig. 3D), the shape of hypoproct rounded apically, not subtriangular (Fig. 3D); shape of heavily sclerotised appendages more round, rounded apically with less distinct dent (Fig. 3C); parameral apodemes long and slightly curved outwards, parameres baring seven setae. Aedeagus more elongate and narrower, stalk-like (Fig. 3A). Gonostylus small, longer than wide apically convex with eight distinct setae subapically before the protruding apical part (Fig. 3B); subapically with a brush of around nine finger-like thick setae.

From *C.orientalis* Søli, it differs in having pectinate antennae, wing vein M_2_ complete, a much shorter posterior fork, absence of an antero-apical organ on fore- and mid-tibia, presence of strong tibial setae and characteristics of the complex terminalia.

From *C.montana*, it differs in the number of long anterior projections on flagellomeres (*C.montana* 1–11, *C.vumanhi* sp. nov. 1–9), colouration of the body (no yellow areas on scutum and lateral thoracic sclerites), presence of strong tibial bristles and details on the terminalia (different shape of T9 without setose medial protuberance and shape of gonostylus, gonocoxites, hypoproct and cerci broader and more rounded without long apical setae).

The new species differs from *C.nigricoxa* in the number and shape of long anterior projections on flagellomeres (in *C.nigricoxa* 1–8, in *C.vumanhi* sp. nov. 1–9), colour patterns of thorax and abdomen, yellow coxae and details on terminalia (different shape of gonostylus, apical dark setae not bent, different shape of T9 without setose medial protuberance, hypoproct and cerci broader and more rounded without long apical setae).

#### Etymology

The new species is named after the Vietnamese acarologist Manh Quang Vu who helped organising the expedition in Tam Dao National Park where the material was collected.

#### Distribution

The species is known only from the type locality in northern Vietnam, but considering the wide distribution of the other species in the genus (Ševčík and Hippa 2010), it is possible that it is present in other localities in the Oriental Region.

#### Notes

During subsequent handling, the specimen sustained additional damage, resulting in detachment of several legs. They are preserved in a separate microvial and include: one fore-leg (comprising femur, tibia and tarsus), two mid-legs (one of which remains partially attached to the specimen, but lacks distal tarsomeres) and two hind legs (one with missing tarsus).

## Identification Keys

### Key to the male species of genus *Chalastonepsia* Søli

**Table:** 

1	Antennae not pectinate, flagellomeres stalked with bead-like apical part, strongly setose. Stem of Cu-fork short. Tibial setae poorly developed. Anteroapical depressed area on fore tibia very shallow with some erect trichia. Small gonostyli without an apical comb of thick setae	*C.orientalis* Søli, 1996
–	Antennae pectinate with conspicuous side projections	2
2	Flagellomeres 1–8 with short, apically darkened side projections. All coxae dark brown. Tibiae without strong bristles. Fore- and mid-tibia without sensory organ. Dark setae on gonostylus slightly bent (figs. 8–9, Ševčík and Hippa 2010). Posterior margin of T9 more straight with medial protuberance less distinct (figs. 10–11, Ševčík and Hippa 2010)	*C.nigricoxa* Ševčík & Hippa, 2010
–	Long flagellar projections on flagellomeres 1–9 or more	3
3	Long flagellar projections on flagellomeres 1–9 only. Point of furcation of CuA beyond base of crossvein r-m. Tibial bristles well developed, mostly arranged in lines with modified bristles, bearing narrow leaf-like lateral processes (fig. 8, Kallweit 1998). No distinct anteroapical tibial organ on fore tibia. T9 medially straight, slightly concave. Hypoproct rounded, not subtriangular. Shape of heavily sclerotised appendages more oval, rounded apically with less distinct dent	*Chalastonepsiavumanhi* sp. nov.
–	Long flagellar projections on flagellomeres 1–10 or more	4
4	Long flagellar projections on flagellomeres 1–10. Point of furcation of CuA very slightly beyond base of crossvein r-m. Tibial bristles well developed with modified bristles not arranged in lines, bearing narrow leaf-like lateral processes. Antero-apical tibial organ on fore-tibia much reduced, untraceable. T9 medially tapering. Hypoproct subtriangular. Shape of heavily sclerotised appendages narrower with more distinct edges, not rounded apically, furrowed, with a distinct dent	*C.hokkaidensis* Kallweit, 1998
–	Long flagellar projections on flagellomeres 1–11. Projection on flagellomere 11 shorter than on flagellomeres 1–10. Yellow areas on scutum and lateral thoracic sclerites. Fore- and mid-tibia without sensory organ. Gonocoxites posteriorly tapering, apical dark setae on gonostylus not bent. Setose medial protuberance on T9 (fig. 12, Ševčík and Hippa (2010))	*C.montana* Ševčík & Hippa, 2010

## Discussion

The study of this fifth species of *Chalastonepsia* contributes to the better knowledge of the genus and confirms that the morphology of the type species *C.orientalis* differs considerably from the other four species and they should probably be placed in a separate genus (Ševčík and Hippa 2010). The new species *C.vumanhi* sp. nov. resembles *C.hokkaidensis* in the structure of the male genitalia and the presence strongly modified thick tibial setae.

Additionally, the species *Chalastonepsia* spec. indet. described by Kallweit (1998) shows more similarities to *C.orientalis* (not pectinate, strongly setose bead-like antennae, short stem of Cu-fork, M_2_ incomplete, structure of the genitalia less complex with small gonostyli without a comb of thick apical bristles) and, thus, could be included in the same genus. As pointed out by Kallweit (1998) and several other authors, these changes in the classification await a taxonomic revision of the Gnoristinae.

Based on the results of a molecular study of the phylogeny of Mycetophilidae (on the position of Metanepsiini, Manotinae and other problematic taxa), *C.orientalis* and *C.nigricoxa* form a well-supported clade (Ševčík et al. 2013), but according to a subsequent study where more material of the genus was analysed, *C.orientalis* was placed in a different clade from *C.nigricoxa*, *C.hokkaidensis* and one undetermined species of *Chalastonepsia*. The molecular phylogeny results do not show a close relationship between *Metanepsia* and *Chalastonepsia* (Ševčík et al. 2013, Kaspřák et al. 2019); hence, the reduced mouthparts which are a common character of the two genera appear to be convergent (Kaspřák et al. 2019).

## Supplementary Material

XML Treatment for Chalastonepsia vumanhi
